# [μ-Bis(di-*o*-tolyl­phosphan­yl)methane-1:2κ^2^ *P*:*P*′]nona­carbonyl-1κ^3^ *C*,2κ^3^ *C*,3κ^3^ *C*-(triphenyl phosphite-3κ*P*)-*triangulo*-triruthenium(0) chloro­form disolvate

**DOI:** 10.1107/S1600536812023227

**Published:** 2012-05-26

**Authors:** Omar bin Shawkataly, Imthyaz Ahmed Khan, Siti Syaida Sirat, Ching Kheng Quah, Hoong-Kun Fun

**Affiliations:** a Chemical Sciences Programme, School of Distance Education, Universiti Sains Malaysia, 11800 USM, Penang, Malaysia; b X-ray Crystallography Unit, School of Physics, Universiti Sains Malaysia, 11800 USM, Penang, Malaysia

## Abstract

In the title solvated *triangulo*-triruthenium compound, [Ru_3_(C_18_H_15_O_3_P)(C_29_H_30_P_2_)(CO)_9_]·2CHCl_3_, the bis­(di-*o*-tolyl­phosphan­yl)methane (dtpm) ligand bridges one of the Ru—Ru bonds and the monodentate phosphine ligand bonds to the third Ru atom. All the P atoms are equatorial with respect to the Ru_3_ triangle: each Ru atom also bears one equatorial and two axial terminal carbonyl ligands. The dihedral angles between the two benzene rings are 75.92 (10) and 78.95 (10)° for the two diphenyl­phosphanyl groups of the dtpm ligand. In the crystal, C—H⋯O hydrogen bonds link the mol­ecules into chains along [010].

## Related literature

For general background to *triangulo*-triruthenium compounds with general formula [Ru_3_(CO)_12-*n*_ *L*_*n*_] (*L* = group 15 ligand) see: Bruce *et al.* (1985 ▶,1988*a* ▶,*b* ▶); Shawkataly *et al.* (1998 ▶, 2004 ▶, 2010 ▶, 2011 ▶). For the preparation of the dtpm ligand, see: Filby *et al.* (2006 ▶). For the stability of the temperature controller used in the data collection, see: Cosier & Glazer (1986 ▶).
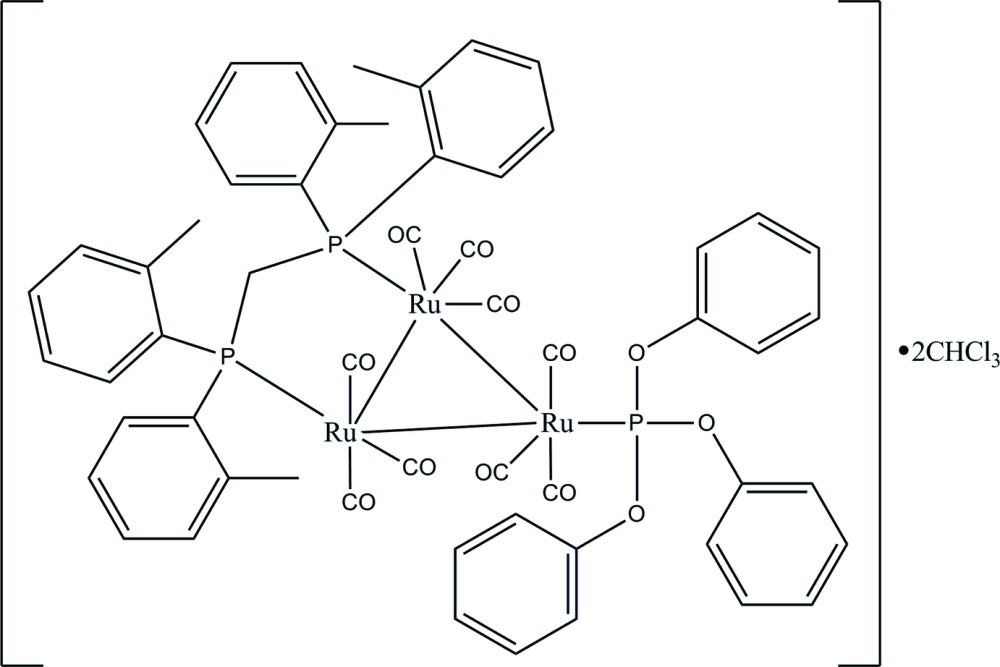


## Experimental

### 

#### Crystal data

[Ru_3_(C_18_H_15_O_3_P)(C_29_H_30_P_2_)(CO)_9_]·2CHCl_3_
*M* *_r_* = 1544.78
Triclinic, 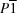

*a* = 12.8185 (4) Å
*b* = 14.9912 (4) Å
*c* = 16.6456 (5) Å
α = 83.374 (1)°
β = 81.380 (1)°
γ = 74.099 (1)°
*V* = 3032.25 (15) Å^3^
*Z* = 2
Mo *K*α radiation
μ = 1.14 mm^−1^
*T* = 100 K
0.46 × 0.30 × 0.21 mm

#### Data collection

Bruker SMART APEXII DUO CCD diffractometer
Absorption correction: multi-scan (*SADABS*; Bruker, 2009 ▶) *T*_min_ = 0.624, *T*_max_ = 0.795
85380 measured reflections
21719 independent reflections
20112 reflections with *I* > 2σ(*I*)
*R*_int_ = 0.019

#### Refinement

*R*[*F* ^2^ > 2σ(*F* ^2^)] = 0.031
*wR*(*F* ^2^) = 0.091
*S* = 1.08
21719 reflections
739 parameters
H-atom parameters constrained
Δρ_max_ = 0.80 e Å^−3^
Δρ_min_ = −2.35 e Å^−3^

### 

Data collection: *APEX2* (Bruker, 2009 ▶); cell refinement: *SAINT* (Bruker, 2009 ▶); data reduction: *SAINT*; program(s) used to solve structure: *SHELXTL* (Sheldrick, 2008 ▶); program(s) used to refine structure: *SHELXTL*; molecular graphics: *SHELXTL*; software used to prepare material for publication: *SHELXTL* and *PLATON* (Spek, 2009 ▶).

## Supplementary Material

Crystal structure: contains datablock(s) global, I. DOI: 10.1107/S1600536812023227/hb6801sup1.cif

Structure factors: contains datablock(s) I. DOI: 10.1107/S1600536812023227/hb6801Isup2.hkl

Additional supplementary materials:  crystallographic information; 3D view; checkCIF report

## Figures and Tables

**Table 1:** Selected bond lengths (Å)

Ru1—P1	2.3476 (5)
Ru2—P2	2.3538 (5)
Ru3—P3	2.2488 (5)

**Table 2:** Hydrogen-bond geometry (Å, °)

*D*—H⋯*A*	*D*—H	H⋯*A*	*D*⋯*A*	*D*—H⋯*A*
C42—H42*A*⋯O1^i^	0.95	2.52	3.384 (3)	151
C56—H56*A*⋯O8^ii^	0.95	2.59	3.343 (3)	136

Symmetry codes: (i) 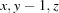; (ii) 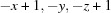.

**Fig. 1.**
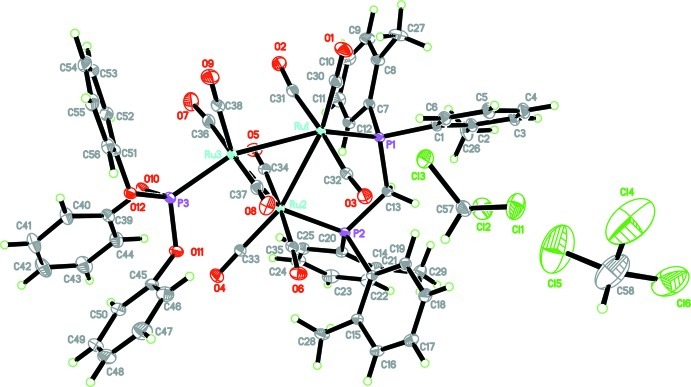
The molecular structure of the title compound showing 50% probability displacement ellipsoids for non-H atoms.

**Fig. 2.**
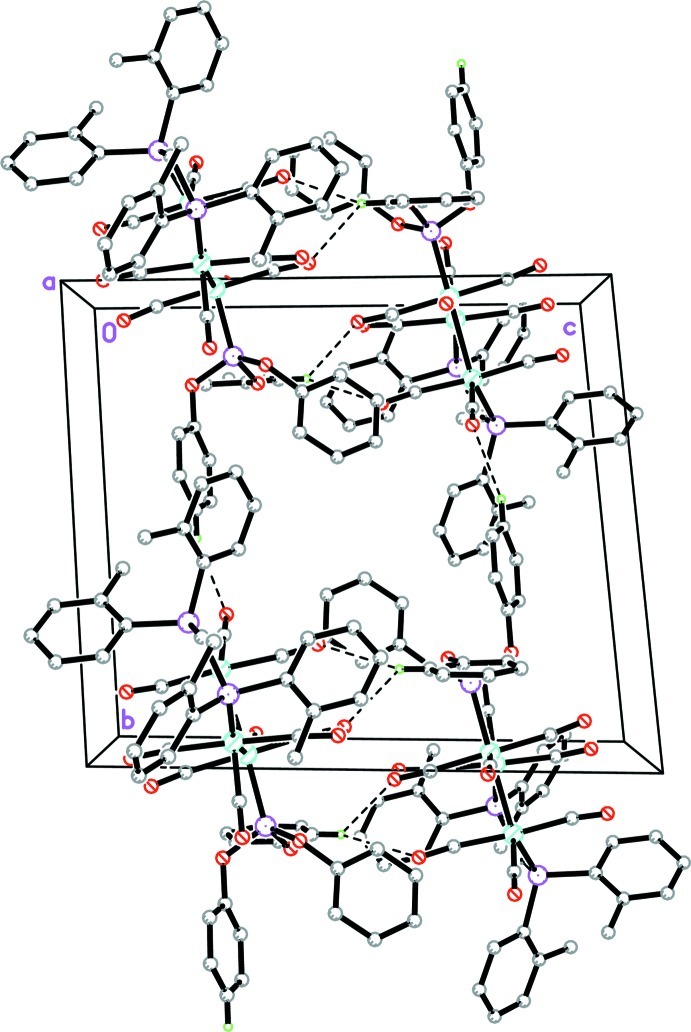
The crystal structure of the title compound, viewed along the a axis. H atoms and solvents molecules not involved in hydrogen bonds (dashed lines) have been omitted for clarity.

## supplementary crystallographic information

### Comment

A large number of substituted derivatives of the type [Ru_3_(CO)_12-_*_n_**L**_n_*] (*L* = group 15 ligand) have been reported (Bruce *et al.*,1985, 1988*a*,*b*). As part of our study on the substitution of transition metal-carbonyl clusters with mixed-ligand complexes, we have published several structures of *triangulo*-triruthenium-carbonyl clusters containing mixed P/As and P/Sb ligands(Shawkataly *et al.*, 1998, 2004, 2010, 2011). Herein we report the synthesis and structure of the title compound.

The asymmetric unit of title triangulo-triruthenium compound consists of one *triangulo*-triruthenium complex molecule and two molecules of chloroform solvent. The bis(di-*o*-tolylphosphanyl)methane ligand bridges the Ru1–Ru2 bond and the monodentate phosphine ligand bonds to the Ru3 atom. Both phosphine ligands are equatorial with respect to the Ru_3_ triangle. Additionally, each Ru atom carries one equatorial and two axial terminal carbonyl ligands (Fig 1). The dihedral angles between the two benzene rings (C1–C6/C7–C12 and C14–C19/C20–C25) are 75.92 (10) and 78.95 (10)° for the two diphenylphosphanyl groups respectively.

In the crystal structure, Fig. 2, molecules are linked *via* C42–H42A···O1 and C56–H56A···O8 hydrogen bonds (Table 1) into one-dimensional chains along [010].

### Experimental

All manipulations were performed under a dry oxygen-free nitrogen atmosphere using standard Schlenk techniques. All solvents were dried over sodium and distilled from sodium benzophenone ketyl under dry oxygen free nitrogen. Triphenylphosphite (BDH) was used as received and bis(di-*o*tolylphosphanyl)methane (Filby *et al.*, 2006) was prepared by reported procedure. Ru_3_(CO)_10_(µ-(2-CH_3_C_6_H_4_)_2_PCH_2_P(2-CH_3_C_6_H_4_)_2_) was prepared by reacting Ru_3_(CO)_12_ with bis(di-*o*-tolylphosphanyl)methane in presence of sodium benzophenone ketyl radical in THF (Shawkataly *et al.*,2011). The title compound was obtained by refluxing equimolar quantities of Ru_3_(CO)_10_(µ-(2-(CH_3_C_6_H_4_)_2_PCH_2_P(2-CH_3_C_6_H_4_)_2_) and triphenylphosphite in hexane under nitrogen atmosphere. Red blocks of the title compound were grown by slow solvent/solvent diffusion of CH_3_OH into CH_2_Cl_2_.

### Refinement

All H atoms were positioned geometrically and refined using a riding model with C–H = 0.95 or 1.00 Å and *U*_iso_(H) = 1.2 or 1.5 *U*_eq_(C).

### Crystal data

**Table:**

[Ru_3_(C_18_H_15_O_3_P)(C_29_H_30_P_2_)(CO)_9_]·2CHCl_3_	*Z* = 2
*M**_r_* = 1544.78	*F*(000) = 1540
Triclinic, *P*1	*D*_x_ = 1.692 Mg m^−^^3^
Hall symbol: -P 1	Mo *K*α radiation, λ = 0.71073 Å
*a* = 12.8185 (4) Å	Cell parameters from 9874 reflections
*b* = 14.9912 (4) Å	θ = 3.2–35.1°
*c* = 16.6456 (5) Å	µ = 1.14 mm^−^^1^
α = 83.374 (1)°	*T* = 100 K
β = 81.380 (1)°	Block, red
γ = 74.099 (1)°	0.46 × 0.30 × 0.21 mm
*V* = 3032.25 (15) Å^3^	

### Data collection

**Table:**

Bruker SMART APEXII DUO CCD diffractometer	21719 independent reflections
Radiation source: fine-focus sealed tube	20112 reflections with *I* > 2σ(*I*)
Graphite monochromator	*R*_int_ = 0.019
φ and ω scans	θ_max_ = 32.5°, θ_min_ = 1.2°
Absorption correction: multi-scan (*SADABS*; Bruker, 2009)	*h* = −19→19
*T*_min_ = 0.624, *T*_max_ = 0.795	*k* = −22→22
85380 measured reflections	*l* = −24→25

### Refinement

**Table:**

Refinement on *F*^2^	Primary atom site location: structure-invariant direct methods
Least-squares matrix: full	Secondary atom site location: difference Fourier map
*R*[*F*^2^ > 2σ(*F*^2^)] = 0.031	Hydrogen site location: inferred from neighbouring sites
*wR*(*F*^2^) = 0.091	H-atom parameters constrained
*S* = 1.08	*w* = 1/[σ^2^(*F*_o_^2^) + (0.0433*P*)^2^ + 5.4575*P*] where *P* = (*F*_o_^2^ + 2*F*_c_^2^)/3
21719 reflections	(Δ/σ)_max_ = 0.001
739 parameters	Δρ_max_ = 0.80 e Å^−^^3^
0 restraints	Δρ_min_ = −2.35 e Å^−^^3^

### Special details

**Table:**

Experimental. The crystal was placed in the cold stream of an Oxford Cryosystems Cobra open-flow nitrogen cryostat (Cosier & Glazer, 1986) operating at 100 K.
Geometry. All esds (except the esd in the dihedral angle between two l.s. planes) are estimated using the full covariance matrix. The cell esds are taken into account individually in the estimation of esds in distances, angles and torsion angles; correlations between esds in cell parameters are only used when they are defined by crystal symmetry. An approximate (isotropic) treatment of cell esds is used for estimating esds involving l.s. planes.
Refinement. Refinement of F^2^ against ALL reflections. The weighted R-factor wR and goodness of fit S are based on F^2^, conventional R-factors R are based on F, with F set to zero for negative F^2^. The threshold expression of F^2^ > 2sigma(F^2^) is used only for calculating R-factors(gt) etc. and is not relevant to the choice of reflections for refinement. R-factors based on F^2^ are statistically about twice as large as those based on F, and R- factors based on ALL data will be even larger.

### Fractional atomic coordinates and isotropic or equivalent isotropic displacement parameters (Å^2^)

**Table:**

	*x*	*y*	*z*	*U*_iso_*/*U*_eq_	
Ru1	0.240263 (11)	0.185552 (9)	0.760162 (8)	0.01124 (3)	
Ru2	0.139490 (11)	0.042574 (9)	0.741319 (8)	0.01144 (3)	
Ru3	0.371742 (11)	0.004225 (9)	0.726241 (9)	0.01257 (3)	
Cl1	0.25562 (6)	0.52361 (5)	0.33076 (5)	0.04271 (16)	
Cl2	0.12538 (5)	0.45637 (5)	0.46909 (5)	0.03657 (13)	
Cl3	0.36034 (5)	0.38965 (4)	0.45163 (5)	0.03466 (13)	
Cl4	0.52852 (14)	0.70175 (18)	−0.02518 (12)	0.1215 (8)	
Cl5	0.42893 (16)	0.57336 (13)	0.07960 (9)	0.1085 (6)	
Cl6	0.29506 (11)	0.73703 (11)	0.00349 (7)	0.0792 (4)	
P1	0.07219 (4)	0.28676 (3)	0.80611 (3)	0.01138 (7)	
P2	−0.03476 (4)	0.14719 (3)	0.73937 (3)	0.01134 (7)	
P3	0.40041 (4)	−0.14531 (3)	0.70099 (3)	0.01292 (8)	
O1	0.41111 (13)	0.29390 (12)	0.74750 (11)	0.0263 (3)	
O2	0.28642 (14)	0.13480 (11)	0.93808 (9)	0.0236 (3)	
O3	0.21740 (13)	0.23920 (11)	0.57851 (9)	0.0214 (3)	
O4	0.08630 (13)	−0.14160 (10)	0.73528 (10)	0.0222 (3)	
O5	0.12618 (15)	0.00889 (12)	0.92782 (10)	0.0251 (3)	
O6	0.16771 (13)	0.05646 (11)	0.55394 (9)	0.0216 (3)	
O7	0.37761 (18)	−0.06511 (13)	0.90726 (10)	0.0329 (4)	
O8	0.39758 (13)	0.06056 (12)	0.54290 (10)	0.0246 (3)	
O9	0.60000 (14)	0.03321 (13)	0.71255 (13)	0.0302 (4)	
O10	0.37818 (12)	−0.20987 (9)	0.78295 (9)	0.0171 (2)	
O11	0.32926 (11)	−0.16811 (10)	0.63892 (9)	0.0175 (2)	
O12	0.51964 (11)	−0.20142 (9)	0.66005 (8)	0.0150 (2)	
C1	0.04446 (15)	0.41116 (12)	0.77021 (11)	0.0142 (3)	
C2	−0.05015 (16)	0.47984 (13)	0.79789 (11)	0.0170 (3)	
C3	−0.06367 (18)	0.57068 (13)	0.76143 (12)	0.0198 (3)	
H3A	−0.1274	0.6173	0.7789	0.024*	
C4	0.01266 (19)	0.59495 (13)	0.70076 (13)	0.0217 (4)	
H4A	0.0013	0.6573	0.6777	0.026*	
C5	0.10578 (18)	0.52763 (14)	0.67388 (12)	0.0204 (3)	
H5A	0.1586	0.5435	0.6324	0.024*	
C6	0.12075 (16)	0.43657 (13)	0.70855 (11)	0.0164 (3)	
H6A	0.1843	0.3905	0.6899	0.020*	
C7	0.04805 (15)	0.28172 (12)	0.91792 (10)	0.0135 (3)	
C8	0.09537 (16)	0.33086 (13)	0.96319 (11)	0.0172 (3)	
C9	0.07724 (18)	0.31933 (15)	1.04842 (12)	0.0219 (4)	
H9A	0.1068	0.3537	1.0795	0.026*	
C10	0.01774 (19)	0.25965 (16)	1.08900 (12)	0.0232 (4)	
H10A	0.0062	0.2538	1.1469	0.028*	
C11	−0.02478 (18)	0.20855 (15)	1.04402 (12)	0.0214 (4)	
H11A	−0.0639	0.1659	1.0710	0.026*	
C12	−0.00992 (16)	0.22004 (13)	0.95941 (11)	0.0161 (3)	
H12A	−0.0398	0.1852	0.9290	0.019*	
C13	−0.05140 (14)	0.26139 (12)	0.77953 (11)	0.0141 (3)	
H13A	−0.0829	0.3107	0.7384	0.017*	
H13B	−0.1057	0.2663	0.8289	0.017*	
C14	−0.06622 (14)	0.17926 (12)	0.63406 (10)	0.0121 (3)	
C15	−0.10205 (14)	0.11996 (12)	0.59087 (11)	0.0139 (3)	
C16	−0.11396 (16)	0.14461 (14)	0.50842 (11)	0.0171 (3)	
H16A	−0.1392	0.1055	0.4791	0.021*	
C17	−0.09008 (16)	0.22440 (14)	0.46811 (11)	0.0190 (3)	
H17A	−0.0989	0.2395	0.4121	0.023*	
C18	−0.05320 (17)	0.28189 (14)	0.51062 (12)	0.0184 (3)	
H18A	−0.0362	0.3365	0.4837	0.022*	
C19	−0.04138 (15)	0.25923 (12)	0.59229 (11)	0.0151 (3)	
H19A	−0.0158	0.2988	0.6208	0.018*	
C20	−0.15337 (15)	0.11352 (13)	0.79839 (10)	0.0147 (3)	
C21	−0.26223 (16)	0.16794 (14)	0.79837 (12)	0.0181 (3)	
C22	−0.34495 (17)	0.13524 (17)	0.84719 (13)	0.0235 (4)	
H22A	−0.4186	0.1711	0.8474	0.028*	
C23	−0.32237 (18)	0.05170 (17)	0.89552 (13)	0.0249 (4)	
H23A	−0.3801	0.0312	0.9281	0.030*	
C24	−0.21559 (18)	−0.00126 (15)	0.89598 (12)	0.0219 (4)	
H24A	−0.1993	−0.0582	0.9290	0.026*	
C25	−0.13201 (16)	0.02985 (14)	0.84741 (11)	0.0177 (3)	
H25A	−0.0587	−0.0067	0.8476	0.021*	
C26	−0.13764 (19)	0.46205 (15)	0.86455 (14)	0.0259 (4)	
H26A	−0.1948	0.5200	0.8730	0.039*	
H26B	−0.1053	0.4393	0.9152	0.039*	
H26C	−0.1696	0.4153	0.8487	0.039*	
C27	0.1639 (2)	0.39520 (17)	0.92475 (13)	0.0258 (4)	
H27A	0.1882	0.4217	0.9675	0.039*	
H27B	0.1204	0.4455	0.8910	0.039*	
H27C	0.2278	0.3604	0.8907	0.039*	
C28	−0.12718 (18)	0.03084 (14)	0.62834 (12)	0.0197 (3)	
H28A	−0.1506	0.0012	0.5872	0.030*	
H28B	−0.1858	0.0444	0.6738	0.030*	
H28C	−0.0616	−0.0111	0.6484	0.030*	
C29	−0.29587 (17)	0.26023 (16)	0.74971 (14)	0.0249 (4)	
H29A	−0.3754	0.2848	0.7595	0.037*	
H29B	−0.2735	0.2517	0.6916	0.037*	
H29C	−0.2605	0.3041	0.7665	0.037*	
C30	0.34552 (15)	0.25359 (13)	0.75230 (12)	0.0165 (3)	
C31	0.26749 (16)	0.14748 (13)	0.87190 (12)	0.0169 (3)	
C32	0.22388 (15)	0.21423 (12)	0.64591 (11)	0.0153 (3)	
C33	0.10801 (15)	−0.07248 (13)	0.73721 (11)	0.0157 (3)	
C34	0.13502 (16)	0.02545 (13)	0.85854 (12)	0.0176 (3)	
C35	0.16153 (15)	0.05443 (13)	0.62333 (11)	0.0158 (3)	
C36	0.36844 (18)	−0.03512 (14)	0.84181 (13)	0.0208 (3)	
C37	0.37983 (15)	0.04360 (13)	0.61124 (12)	0.0173 (3)	
C38	0.51501 (17)	0.02119 (14)	0.72039 (13)	0.0201 (3)	
C39	0.37275 (16)	−0.30190 (13)	0.78418 (12)	0.0173 (3)	
C40	0.46430 (18)	−0.37270 (14)	0.76288 (14)	0.0233 (4)	
H40A	0.5336	−0.3604	0.7469	0.028*	
C41	0.4522 (2)	−0.46287 (16)	0.76544 (17)	0.0306 (5)	
H41A	0.5138	−0.5123	0.7499	0.037*	
C42	0.3509 (2)	−0.48106 (16)	0.79045 (18)	0.0337 (5)	
H42A	0.3435	−0.5426	0.7919	0.040*	
C43	0.2613 (2)	−0.40921 (17)	0.81314 (17)	0.0304 (5)	
H43A	0.1925	−0.4217	0.8312	0.036*	
C44	0.27115 (18)	−0.31881 (15)	0.80980 (14)	0.0223 (4)	
H44A	0.2093	−0.2693	0.8248	0.027*	
C45	0.36400 (15)	−0.22577 (13)	0.57396 (12)	0.0169 (3)	
C46	0.40476 (19)	−0.18904 (16)	0.49954 (13)	0.0240 (4)	
H46A	0.4138	−0.1277	0.4937	0.029*	
C47	0.4322 (2)	−0.2435 (2)	0.43359 (15)	0.0326 (5)	
H47A	0.4604	−0.2194	0.3821	0.039*	
C48	0.4184 (2)	−0.33311 (19)	0.44246 (16)	0.0340 (5)	
H48A	0.4380	−0.3704	0.3973	0.041*	
C49	0.3764 (2)	−0.36781 (16)	0.51690 (16)	0.0293 (5)	
H49A	0.3665	−0.4288	0.5225	0.035*	
C50	0.34808 (18)	−0.31425 (15)	0.58425 (14)	0.0220 (4)	
H50A	0.3188	−0.3379	0.6355	0.026*	
C51	0.61667 (14)	−0.19515 (12)	0.68423 (11)	0.0144 (3)	
C52	0.63069 (16)	−0.19898 (13)	0.76580 (12)	0.0181 (3)	
H52A	0.5743	−0.2075	0.8076	0.022*	
C53	0.72967 (18)	−0.19007 (14)	0.78498 (14)	0.0221 (4)	
H53A	0.7401	−0.1911	0.8404	0.026*	
C54	0.81255 (17)	−0.17980 (14)	0.72407 (15)	0.0234 (4)	
H54A	0.8792	−0.1730	0.7377	0.028*	
C55	0.79825 (16)	−0.17942 (14)	0.64243 (14)	0.0218 (4)	
H55A	0.8561	−0.1744	0.6007	0.026*	
C56	0.69940 (16)	−0.18638 (13)	0.62196 (12)	0.0175 (3)	
H56A	0.6888	−0.1851	0.5665	0.021*	
C57	0.24503 (17)	0.42747 (15)	0.40016 (15)	0.0235 (4)	
H57A	0.2411	0.3757	0.3688	0.028*	
C58	0.4194 (4)	0.6485 (4)	−0.0080 (2)	0.0709 (13)	
H58A	0.4210	0.6131	−0.0557	0.085*	

### Atomic displacement parameters (Å^2^)

**Table:**

	*U*^11^	*U*^22^	*U*^33^	*U*^12^	*U*^13^	*U*^23^
Ru1	0.01275 (6)	0.01008 (6)	0.01143 (6)	−0.00347 (4)	−0.00121 (4)	−0.00222 (4)
Ru2	0.01249 (6)	0.01028 (6)	0.01241 (6)	−0.00334 (4)	−0.00257 (4)	−0.00223 (4)
Ru3	0.01249 (6)	0.01098 (6)	0.01448 (6)	−0.00259 (4)	−0.00173 (4)	−0.00314 (4)
Cl1	0.0345 (3)	0.0383 (3)	0.0509 (4)	−0.0095 (3)	−0.0052 (3)	0.0150 (3)
Cl2	0.0243 (2)	0.0383 (3)	0.0438 (3)	−0.0061 (2)	0.0048 (2)	−0.0070 (3)
Cl3	0.0258 (2)	0.0271 (3)	0.0505 (4)	0.0004 (2)	−0.0141 (2)	−0.0061 (2)
Cl4	0.0678 (9)	0.197 (2)	0.0928 (11)	−0.0450 (11)	−0.0087 (8)	0.0391 (13)
Cl5	0.1206 (13)	0.1155 (12)	0.0541 (7)	0.0044 (10)	0.0090 (7)	0.0261 (7)
Cl6	0.0680 (7)	0.1084 (10)	0.0539 (6)	0.0018 (6)	−0.0162 (5)	−0.0267 (6)
P1	0.01406 (18)	0.01094 (17)	0.00978 (17)	−0.00400 (14)	−0.00118 (13)	−0.00201 (13)
P2	0.01271 (17)	0.01163 (17)	0.01051 (17)	−0.00378 (14)	−0.00206 (13)	−0.00194 (13)
P3	0.01289 (18)	0.01228 (18)	0.01419 (18)	−0.00377 (14)	−0.00097 (14)	−0.00325 (14)
O1	0.0208 (7)	0.0253 (7)	0.0365 (9)	−0.0115 (6)	0.0002 (6)	−0.0086 (6)
O2	0.0312 (8)	0.0220 (7)	0.0175 (6)	−0.0054 (6)	−0.0069 (6)	−0.0004 (5)
O3	0.0283 (7)	0.0202 (6)	0.0151 (6)	−0.0054 (5)	−0.0024 (5)	−0.0013 (5)
O4	0.0239 (7)	0.0159 (6)	0.0293 (8)	−0.0082 (5)	−0.0047 (6)	−0.0024 (5)
O5	0.0332 (8)	0.0286 (8)	0.0169 (6)	−0.0125 (6)	−0.0068 (6)	0.0007 (5)
O6	0.0234 (7)	0.0274 (7)	0.0159 (6)	−0.0091 (6)	−0.0004 (5)	−0.0065 (5)
O7	0.0486 (11)	0.0265 (8)	0.0182 (7)	0.0019 (7)	−0.0082 (7)	−0.0035 (6)
O8	0.0216 (7)	0.0272 (7)	0.0204 (7)	−0.0019 (6)	0.0011 (5)	0.0018 (6)
O9	0.0205 (7)	0.0282 (8)	0.0461 (10)	−0.0107 (6)	−0.0064 (7)	−0.0070 (7)
O10	0.0228 (6)	0.0133 (5)	0.0161 (6)	−0.0073 (5)	0.0004 (5)	−0.0026 (4)
O11	0.0145 (6)	0.0200 (6)	0.0196 (6)	−0.0040 (5)	−0.0024 (5)	−0.0086 (5)
O12	0.0125 (5)	0.0163 (6)	0.0169 (6)	−0.0034 (4)	−0.0015 (4)	−0.0055 (4)
C1	0.0195 (7)	0.0113 (6)	0.0121 (7)	−0.0037 (6)	−0.0026 (6)	−0.0016 (5)
C2	0.0217 (8)	0.0135 (7)	0.0144 (7)	−0.0023 (6)	−0.0006 (6)	−0.0032 (6)
C3	0.0269 (9)	0.0127 (7)	0.0179 (8)	−0.0013 (6)	−0.0030 (7)	−0.0021 (6)
C4	0.0324 (10)	0.0132 (7)	0.0187 (8)	−0.0051 (7)	−0.0048 (7)	0.0014 (6)
C5	0.0261 (9)	0.0168 (8)	0.0178 (8)	−0.0075 (7)	−0.0007 (7)	0.0022 (6)
C6	0.0196 (8)	0.0144 (7)	0.0152 (7)	−0.0051 (6)	−0.0008 (6)	−0.0008 (6)
C7	0.0170 (7)	0.0134 (7)	0.0105 (6)	−0.0045 (6)	−0.0014 (5)	−0.0017 (5)
C8	0.0218 (8)	0.0190 (8)	0.0128 (7)	−0.0082 (6)	−0.0024 (6)	−0.0026 (6)
C9	0.0288 (10)	0.0268 (9)	0.0127 (7)	−0.0102 (8)	−0.0039 (7)	−0.0037 (7)
C10	0.0288 (10)	0.0278 (10)	0.0123 (7)	−0.0077 (8)	−0.0015 (7)	0.0003 (7)
C11	0.0246 (9)	0.0231 (9)	0.0155 (8)	−0.0077 (7)	0.0007 (7)	0.0019 (6)
C12	0.0190 (8)	0.0158 (7)	0.0143 (7)	−0.0066 (6)	−0.0006 (6)	−0.0012 (6)
C13	0.0145 (7)	0.0134 (7)	0.0145 (7)	−0.0021 (5)	−0.0028 (5)	−0.0043 (5)
C14	0.0125 (6)	0.0128 (6)	0.0112 (6)	−0.0036 (5)	−0.0017 (5)	−0.0016 (5)
C15	0.0151 (7)	0.0152 (7)	0.0126 (7)	−0.0052 (6)	−0.0021 (5)	−0.0021 (5)
C16	0.0187 (8)	0.0206 (8)	0.0140 (7)	−0.0071 (6)	−0.0039 (6)	−0.0021 (6)
C17	0.0208 (8)	0.0244 (9)	0.0128 (7)	−0.0072 (7)	−0.0041 (6)	0.0008 (6)
C18	0.0220 (8)	0.0184 (8)	0.0153 (7)	−0.0071 (6)	−0.0035 (6)	0.0029 (6)
C19	0.0168 (7)	0.0145 (7)	0.0146 (7)	−0.0052 (6)	−0.0023 (6)	−0.0005 (5)
C20	0.0158 (7)	0.0184 (7)	0.0111 (7)	−0.0069 (6)	−0.0005 (5)	−0.0016 (5)
C21	0.0159 (7)	0.0234 (8)	0.0154 (7)	−0.0062 (6)	−0.0011 (6)	−0.0020 (6)
C22	0.0176 (8)	0.0331 (11)	0.0207 (9)	−0.0101 (7)	0.0022 (7)	−0.0033 (7)
C23	0.0237 (9)	0.0358 (11)	0.0184 (9)	−0.0164 (8)	0.0025 (7)	−0.0012 (8)
C24	0.0270 (9)	0.0255 (9)	0.0159 (8)	−0.0134 (8)	−0.0013 (7)	0.0018 (7)
C25	0.0214 (8)	0.0195 (8)	0.0137 (7)	−0.0086 (6)	−0.0016 (6)	−0.0002 (6)
C26	0.0287 (10)	0.0173 (8)	0.0248 (10)	−0.0002 (7)	0.0090 (8)	−0.0036 (7)
C27	0.0365 (11)	0.0321 (11)	0.0174 (8)	−0.0230 (9)	−0.0036 (8)	−0.0029 (7)
C28	0.0281 (9)	0.0197 (8)	0.0163 (8)	−0.0139 (7)	−0.0049 (7)	−0.0006 (6)
C29	0.0150 (8)	0.0279 (10)	0.0286 (10)	−0.0030 (7)	−0.0025 (7)	0.0038 (8)
C30	0.0161 (7)	0.0148 (7)	0.0186 (8)	−0.0035 (6)	−0.0008 (6)	−0.0039 (6)
C31	0.0195 (8)	0.0141 (7)	0.0177 (8)	−0.0048 (6)	−0.0028 (6)	−0.0013 (6)
C32	0.0172 (7)	0.0141 (7)	0.0153 (7)	−0.0045 (6)	−0.0010 (6)	−0.0038 (6)
C33	0.0155 (7)	0.0148 (7)	0.0172 (7)	−0.0040 (6)	−0.0026 (6)	−0.0023 (6)
C34	0.0199 (8)	0.0164 (7)	0.0184 (8)	−0.0059 (6)	−0.0058 (6)	−0.0011 (6)
C35	0.0152 (7)	0.0157 (7)	0.0176 (8)	−0.0051 (6)	−0.0016 (6)	−0.0041 (6)
C36	0.0252 (9)	0.0168 (8)	0.0190 (8)	−0.0006 (7)	−0.0042 (7)	−0.0054 (6)
C37	0.0141 (7)	0.0154 (7)	0.0212 (8)	−0.0019 (6)	−0.0014 (6)	−0.0018 (6)
C38	0.0197 (8)	0.0164 (8)	0.0259 (9)	−0.0049 (6)	−0.0050 (7)	−0.0049 (7)
C39	0.0220 (8)	0.0140 (7)	0.0165 (7)	−0.0067 (6)	−0.0001 (6)	−0.0019 (6)
C40	0.0233 (9)	0.0163 (8)	0.0279 (10)	−0.0039 (7)	0.0023 (7)	−0.0027 (7)
C41	0.0344 (12)	0.0154 (8)	0.0381 (12)	−0.0043 (8)	0.0045 (10)	−0.0037 (8)
C42	0.0422 (14)	0.0174 (9)	0.0433 (14)	−0.0143 (9)	0.0017 (11)	−0.0023 (9)
C43	0.0334 (11)	0.0235 (10)	0.0374 (12)	−0.0161 (9)	0.0000 (9)	0.0010 (9)
C44	0.0218 (9)	0.0188 (8)	0.0259 (9)	−0.0077 (7)	0.0007 (7)	0.0006 (7)
C45	0.0152 (7)	0.0183 (8)	0.0182 (8)	−0.0032 (6)	−0.0041 (6)	−0.0062 (6)
C46	0.0261 (9)	0.0277 (10)	0.0194 (9)	−0.0084 (8)	−0.0016 (7)	−0.0050 (7)
C47	0.0369 (12)	0.0407 (13)	0.0189 (9)	−0.0067 (10)	−0.0015 (9)	−0.0081 (9)
C48	0.0387 (13)	0.0361 (12)	0.0266 (11)	0.0007 (10)	−0.0101 (9)	−0.0180 (9)
C49	0.0359 (12)	0.0207 (9)	0.0343 (12)	−0.0033 (8)	−0.0143 (9)	−0.0112 (8)
C50	0.0250 (9)	0.0196 (8)	0.0243 (9)	−0.0071 (7)	−0.0069 (7)	−0.0052 (7)
C51	0.0135 (7)	0.0113 (6)	0.0182 (7)	−0.0021 (5)	−0.0017 (6)	−0.0032 (5)
C52	0.0186 (8)	0.0175 (8)	0.0178 (8)	−0.0032 (6)	−0.0033 (6)	−0.0019 (6)
C53	0.0223 (9)	0.0195 (8)	0.0256 (9)	−0.0030 (7)	−0.0109 (7)	−0.0022 (7)
C54	0.0168 (8)	0.0175 (8)	0.0374 (11)	−0.0046 (6)	−0.0095 (8)	0.0004 (7)
C55	0.0150 (8)	0.0167 (8)	0.0326 (10)	−0.0047 (6)	−0.0008 (7)	0.0007 (7)
C56	0.0171 (7)	0.0142 (7)	0.0201 (8)	−0.0035 (6)	−0.0002 (6)	−0.0011 (6)
C57	0.0203 (9)	0.0189 (8)	0.0315 (10)	−0.0034 (7)	−0.0034 (7)	−0.0066 (7)
C58	0.058 (2)	0.106 (4)	0.0365 (18)	−0.009 (2)	−0.0053 (16)	0.014 (2)

### Geometric parameters (Å, º)

**Table:**

Ru1—C30	1.8837 (19)	C15—C16	1.399 (2)
Ru1—C32	1.9306 (18)	C15—C28	1.504 (3)
Ru1—C31	1.9359 (19)	C16—C17	1.389 (3)
Ru1—P1	2.3476 (5)	C16—H16A	0.9500
Ru1—Ru3	2.8473 (2)	C17—C18	1.390 (3)
Ru1—Ru2	2.8557 (2)	C17—H17A	0.9500
Ru2—C33	1.8867 (18)	C18—C19	1.383 (3)
Ru2—C34	1.9328 (19)	C18—H18A	0.9500
Ru2—C35	1.9363 (19)	C19—H19A	0.9500
Ru2—P2	2.3538 (5)	C20—C25	1.399 (3)
Ru2—Ru3	2.8510 (2)	C20—C21	1.410 (3)
Ru3—C38	1.909 (2)	C21—C22	1.400 (3)
Ru3—C37	1.933 (2)	C21—C29	1.508 (3)
Ru3—C36	1.944 (2)	C22—C23	1.392 (3)
Ru3—P3	2.2488 (5)	C22—H22A	0.9500
Cl1—C57	1.762 (2)	C23—C24	1.382 (3)
Cl2—C57	1.755 (2)	C23—H23A	0.9500
Cl3—C57	1.746 (2)	C24—C25	1.395 (3)
Cl4—C58	1.765 (6)	C24—H24A	0.9500
Cl5—C58	1.734 (4)	C25—H25A	0.9500
Cl6—C58	1.774 (5)	C26—H26A	0.9800
P1—C7	1.8365 (17)	C26—H26B	0.9800
P1—C1	1.8452 (18)	C26—H26C	0.9800
P1—C13	1.8519 (18)	C27—H27A	0.9800
P2—C14	1.8379 (17)	C27—H27B	0.9800
P2—C20	1.8412 (18)	C27—H27C	0.9800
P2—C13	1.8560 (17)	C28—H28A	0.9800
P3—O11	1.5972 (14)	C28—H28B	0.9800
P3—O10	1.6153 (15)	C28—H28C	0.9800
P3—O12	1.6196 (14)	C29—H29A	0.9800
O1—C30	1.151 (2)	C29—H29B	0.9800
O2—C31	1.147 (2)	C29—H29C	0.9800
O3—C32	1.149 (2)	C39—C40	1.382 (3)
O4—C33	1.149 (2)	C39—C44	1.390 (3)
O5—C34	1.147 (2)	C40—C41	1.397 (3)
O6—C35	1.143 (2)	C40—H40A	0.9500
O7—C36	1.140 (3)	C41—C42	1.393 (4)
O8—C37	1.139 (3)	C41—H41A	0.9500
O9—C38	1.138 (3)	C42—C43	1.381 (4)
O10—C39	1.398 (2)	C42—H42A	0.9500
O11—C45	1.405 (2)	C43—C44	1.389 (3)
O12—C51	1.393 (2)	C43—H43A	0.9500
C1—C6	1.403 (3)	C44—H44A	0.9500
C1—C2	1.415 (3)	C45—C50	1.384 (3)
C2—C3	1.401 (3)	C45—C46	1.385 (3)
C2—C26	1.511 (3)	C46—C47	1.389 (3)
C3—C4	1.387 (3)	C46—H46A	0.9500
C3—H3A	0.9500	C47—C48	1.391 (4)
C4—C5	1.388 (3)	C47—H47A	0.9500
C4—H4A	0.9500	C48—C49	1.379 (4)
C5—C6	1.393 (3)	C48—H48A	0.9500
C5—H5A	0.9500	C49—C50	1.400 (3)
C6—H6A	0.9500	C49—H49A	0.9500
C7—C12	1.400 (2)	C50—H50A	0.9500
C7—C8	1.410 (2)	C51—C52	1.389 (3)
C8—C9	1.401 (3)	C51—C56	1.390 (3)
C8—C27	1.505 (3)	C52—C53	1.398 (3)
C9—C10	1.385 (3)	C52—H52A	0.9500
C9—H9A	0.9500	C53—C54	1.382 (3)
C10—C11	1.389 (3)	C53—H53A	0.9500
C10—H10A	0.9500	C54—C55	1.397 (3)
C11—C12	1.390 (3)	C54—H54A	0.9500
C11—H11A	0.9500	C55—C56	1.394 (3)
C12—H12A	0.9500	C55—H55A	0.9500
C13—H13A	0.9900	C56—H56A	0.9500
C13—H13B	0.9900	C57—H57A	1.0000
C14—C19	1.404 (2)	C58—H58A	1.0000
C14—C15	1.410 (2)		
			
C30—Ru1—C32	91.68 (8)	C17—C18—H18A	120.1
C30—Ru1—C31	87.98 (8)	C18—C19—C14	121.66 (17)
C32—Ru1—C31	174.89 (8)	C18—C19—H19A	119.2
C30—Ru1—P1	106.24 (6)	C14—C19—H19A	119.2
C32—Ru1—P1	95.01 (6)	C25—C20—C21	119.08 (17)
C31—Ru1—P1	89.99 (6)	C25—C20—P2	116.96 (14)
C30—Ru1—Ru3	102.18 (6)	C21—C20—P2	123.94 (14)
C32—Ru1—Ru3	89.88 (5)	C22—C21—C20	118.31 (19)
C31—Ru1—Ru3	85.21 (6)	C22—C21—C29	117.39 (18)
P1—Ru1—Ru3	150.984 (12)	C20—C21—C29	124.29 (17)
C30—Ru1—Ru2	160.93 (6)	C23—C22—C21	121.9 (2)
C32—Ru1—Ru2	82.18 (5)	C23—C22—H22A	119.0
C31—Ru1—Ru2	96.54 (6)	C21—C22—H22A	119.0
P1—Ru1—Ru2	92.322 (12)	C24—C23—C22	119.74 (19)
Ru3—Ru1—Ru2	59.989 (5)	C24—C23—H23A	120.1
C33—Ru2—C34	91.82 (8)	C22—C23—H23A	120.1
C33—Ru2—C35	88.96 (8)	C23—C24—C25	119.24 (19)
C34—Ru2—C35	173.59 (8)	C23—C24—H24A	120.4
C33—Ru2—P2	101.70 (6)	C25—C24—H24A	120.4
C34—Ru2—P2	96.36 (6)	C24—C25—C20	121.71 (19)
C35—Ru2—P2	89.70 (6)	C24—C25—H25A	119.1
C33—Ru2—Ru3	106.16 (6)	C20—C25—H25A	119.1
C34—Ru2—Ru3	89.61 (6)	C2—C26—H26A	109.5
C35—Ru2—Ru3	84.07 (5)	C2—C26—H26B	109.5
P2—Ru2—Ru3	151.297 (12)	H26A—C26—H26B	109.5
C33—Ru2—Ru1	164.78 (6)	C2—C26—H26C	109.5
C34—Ru2—Ru1	82.59 (6)	H26A—C26—H26C	109.5
C35—Ru2—Ru1	95.06 (5)	H26B—C26—H26C	109.5
P2—Ru2—Ru1	93.013 (12)	C8—C27—H27A	109.5
Ru3—Ru2—Ru1	59.860 (5)	C8—C27—H27B	109.5
C38—Ru3—C37	86.27 (9)	H27A—C27—H27B	109.5
C38—Ru3—C36	91.88 (9)	C8—C27—H27C	109.5
C37—Ru3—C36	178.14 (9)	H27A—C27—H27C	109.5
C38—Ru3—P3	104.36 (6)	H27B—C27—H27C	109.5
C37—Ru3—P3	91.72 (6)	C15—C28—H28A	109.5
C36—Ru3—P3	88.63 (6)	C15—C28—H28B	109.5
C38—Ru3—Ru1	101.06 (6)	H28A—C28—H28B	109.5
C37—Ru3—Ru1	89.13 (5)	C15—C28—H28C	109.5
C36—Ru3—Ru1	91.34 (6)	H28A—C28—H28C	109.5
P3—Ru3—Ru1	154.566 (13)	H28B—C28—H28C	109.5
C38—Ru3—Ru2	161.16 (6)	C21—C29—H29A	109.5
C37—Ru3—Ru2	91.74 (6)	C21—C29—H29B	109.5
C36—Ru3—Ru2	90.06 (6)	H29A—C29—H29B	109.5
P3—Ru3—Ru2	94.414 (13)	C21—C29—H29C	109.5
Ru1—Ru3—Ru2	60.152 (5)	H29A—C29—H29C	109.5
C7—P1—C1	106.45 (8)	H29B—C29—H29C	109.5
C7—P1—C13	102.54 (8)	O1—C30—Ru1	178.93 (18)
C1—P1—C13	99.39 (8)	O2—C31—Ru1	171.92 (17)
C7—P1—Ru1	111.41 (6)	O3—C32—Ru1	173.12 (16)
C1—P1—Ru1	119.03 (6)	O4—C33—Ru2	178.39 (17)
C13—P1—Ru1	116.12 (6)	O5—C34—Ru2	172.95 (17)
C14—P2—C20	107.18 (8)	O6—C35—Ru2	173.54 (16)
C14—P2—C13	103.10 (8)	O7—C36—Ru3	172.26 (19)
C20—P2—C13	100.03 (8)	O8—C37—Ru3	171.62 (17)
C14—P2—Ru2	110.49 (6)	O9—C38—Ru3	176.2 (2)
C20—P2—Ru2	118.78 (6)	C40—C39—C44	121.77 (18)
C13—P2—Ru2	115.67 (6)	C40—C39—O10	121.64 (18)
O11—P3—O10	104.17 (8)	C44—C39—O10	116.58 (17)
O11—P3—O12	97.81 (7)	C39—C40—C41	118.3 (2)
O10—P3—O12	103.38 (8)	C39—C40—H40A	120.9
O11—P3—Ru3	117.80 (6)	C41—C40—H40A	120.9
O10—P3—Ru3	111.92 (5)	C42—C41—C40	120.8 (2)
O12—P3—Ru3	119.50 (5)	C42—C41—H41A	119.6
C39—O10—P3	124.36 (12)	C40—C41—H41A	119.6
C45—O11—P3	128.76 (12)	C43—C42—C41	119.7 (2)
C51—O12—P3	122.70 (11)	C43—C42—H42A	120.2
C6—C1—C2	119.10 (16)	C41—C42—H42A	120.2
C6—C1—P1	116.59 (13)	C42—C43—C44	120.5 (2)
C2—C1—P1	124.23 (14)	C42—C43—H43A	119.7
C3—C2—C1	117.98 (18)	C44—C43—H43A	119.7
C3—C2—C26	117.62 (17)	C43—C44—C39	119.0 (2)
C1—C2—C26	124.40 (17)	C43—C44—H44A	120.5
C4—C3—C2	122.33 (18)	C39—C44—H44A	120.5
C4—C3—H3A	118.8	C50—C45—C46	122.09 (19)
C2—C3—H3A	118.8	C50—C45—O11	119.24 (18)
C3—C4—C5	119.68 (18)	C46—C45—O11	118.45 (18)
C3—C4—H4A	120.2	C45—C46—C47	118.8 (2)
C5—C4—H4A	120.2	C45—C46—H46A	120.6
C4—C5—C6	119.17 (18)	C47—C46—H46A	120.6
C4—C5—H5A	120.4	C46—C47—C48	120.3 (2)
C6—C5—H5A	120.4	C46—C47—H47A	119.8
C5—C6—C1	121.74 (18)	C48—C47—H47A	119.8
C5—C6—H6A	119.1	C49—C48—C47	119.9 (2)
C1—C6—H6A	119.1	C49—C48—H48A	120.1
C12—C7—C8	119.03 (16)	C47—C48—H48A	120.1
C12—C7—P1	118.72 (13)	C48—C49—C50	120.8 (2)
C8—C7—P1	121.94 (14)	C48—C49—H49A	119.6
C9—C8—C7	118.19 (17)	C50—C49—H49A	119.6
C9—C8—C27	118.36 (17)	C45—C50—C49	118.1 (2)
C7—C8—C27	123.44 (17)	C45—C50—H50A	121.0
C10—C9—C8	122.32 (19)	C49—C50—H50A	121.0
C10—C9—H9A	118.8	C52—C51—C56	121.87 (17)
C8—C9—H9A	118.8	C52—C51—O12	122.02 (17)
C9—C10—C11	119.15 (18)	C56—C51—O12	116.11 (16)
C9—C10—H10A	120.4	C51—C52—C53	118.50 (19)
C11—C10—H10A	120.4	C51—C52—H52A	120.8
C10—C11—C12	119.71 (19)	C53—C52—H52A	120.8
C10—C11—H11A	120.1	C54—C53—C52	120.7 (2)
C12—C11—H11A	120.1	C54—C53—H53A	119.7
C11—C12—C7	121.50 (17)	C52—C53—H53A	119.7
C11—C12—H12A	119.2	C53—C54—C55	119.96 (18)
C7—C12—H12A	119.2	C53—C54—H54A	120.0
P1—C13—P2	117.58 (9)	C55—C54—H54A	120.0
P1—C13—H13A	107.9	C56—C55—C54	120.26 (19)
P2—C13—H13A	107.9	C56—C55—H55A	119.9
P1—C13—H13B	107.9	C54—C55—H55A	119.9
P2—C13—H13B	107.9	C51—C56—C55	118.70 (19)
H13A—C13—H13B	107.2	C51—C56—H56A	120.6
C19—C14—C15	118.84 (16)	C55—C56—H56A	120.6
C19—C14—P2	119.06 (13)	Cl3—C57—Cl2	110.86 (13)
C15—C14—P2	121.65 (13)	Cl3—C57—Cl1	110.23 (12)
C16—C15—C14	118.45 (16)	Cl2—C57—Cl1	110.37 (12)
C16—C15—C28	117.97 (16)	Cl3—C57—H57A	108.4
C14—C15—C28	123.58 (16)	Cl2—C57—H57A	108.4
C17—C16—C15	122.06 (17)	Cl1—C57—H57A	108.4
C17—C16—H16A	119.0	Cl5—C58—Cl4	110.6 (3)
C15—C16—H16A	119.0	Cl5—C58—Cl6	109.4 (2)
C16—C17—C18	119.28 (17)	Cl4—C58—Cl6	108.3 (3)
C16—C17—H17A	120.4	Cl5—C58—H58A	109.5
C18—C17—H17A	120.4	Cl4—C58—H58A	109.5
C19—C18—C17	119.70 (17)	Cl6—C58—H58A	109.5
C19—C18—H18A	120.1		
			
C30—Ru1—Ru2—C33	47.0 (3)	C2—C3—C4—C5	0.6 (3)
C32—Ru1—Ru2—C33	119.1 (2)	C3—C4—C5—C6	0.0 (3)
C31—Ru1—Ru2—C33	−55.9 (2)	C4—C5—C6—C1	−0.3 (3)
P1—Ru1—Ru2—C33	−146.1 (2)	C2—C1—C6—C5	0.0 (3)
Ru3—Ru1—Ru2—C33	24.7 (2)	P1—C1—C6—C5	176.86 (15)
C30—Ru1—Ru2—C34	116.15 (19)	C1—P1—C7—C12	136.24 (15)
C32—Ru1—Ru2—C34	−171.72 (8)	C13—P1—C7—C12	32.35 (16)
C31—Ru1—Ru2—C34	13.27 (8)	Ru1—P1—C7—C12	−92.52 (15)
P1—Ru1—Ru2—C34	−76.98 (6)	C1—P1—C7—C8	−50.30 (17)
Ru3—Ru1—Ru2—C34	93.87 (6)	C13—P1—C7—C8	−154.19 (16)
C30—Ru1—Ru2—C35	−57.86 (19)	Ru1—P1—C7—C8	80.94 (16)
C32—Ru1—Ru2—C35	14.28 (8)	C12—C7—C8—C9	−3.3 (3)
C31—Ru1—Ru2—C35	−160.73 (8)	P1—C7—C8—C9	−176.75 (15)
P1—Ru1—Ru2—C35	109.01 (6)	C12—C7—C8—C27	176.9 (2)
Ru3—Ru1—Ru2—C35	−80.13 (6)	P1—C7—C8—C27	3.5 (3)
C30—Ru1—Ru2—P2	−147.82 (18)	C7—C8—C9—C10	2.0 (3)
C32—Ru1—Ru2—P2	−75.69 (6)	C27—C8—C9—C10	−178.2 (2)
C31—Ru1—Ru2—P2	109.30 (6)	C8—C9—C10—C11	0.7 (3)
P1—Ru1—Ru2—P2	19.050 (16)	C9—C10—C11—C12	−1.9 (3)
Ru3—Ru1—Ru2—P2	−170.096 (12)	C10—C11—C12—C7	0.5 (3)
C30—Ru1—Ru2—Ru3	22.28 (18)	C8—C7—C12—C11	2.1 (3)
C32—Ru1—Ru2—Ru3	94.41 (6)	P1—C7—C12—C11	175.78 (16)
C31—Ru1—Ru2—Ru3	−80.60 (6)	C7—P1—C13—P2	−109.17 (11)
P1—Ru1—Ru2—Ru3	−170.855 (12)	C1—P1—C13—P2	141.51 (10)
C30—Ru1—Ru3—C38	5.81 (9)	Ru1—P1—C13—P2	12.54 (12)
C32—Ru1—Ru3—C38	97.50 (9)	C14—P2—C13—P1	−114.17 (11)
C31—Ru1—Ru3—C38	−81.08 (9)	C20—P2—C13—P1	135.39 (11)
P1—Ru1—Ru3—C38	−162.36 (7)	Ru2—P2—C13—P1	6.53 (12)
Ru2—Ru1—Ru3—C38	178.53 (7)	C20—P2—C14—C19	136.37 (14)
C30—Ru1—Ru3—C37	−80.22 (8)	C13—P2—C14—C19	31.34 (16)
C32—Ru1—Ru3—C37	11.47 (8)	Ru2—P2—C14—C19	−92.83 (14)
C31—Ru1—Ru3—C37	−167.10 (8)	C20—P2—C14—C15	−51.51 (16)
P1—Ru1—Ru3—C37	111.61 (6)	C13—P2—C14—C15	−156.53 (14)
Ru2—Ru1—Ru3—C37	92.50 (6)	Ru2—P2—C14—C15	79.29 (15)
C30—Ru1—Ru3—C36	97.98 (9)	C19—C14—C15—C16	−1.6 (3)
C32—Ru1—Ru3—C36	−170.32 (9)	P2—C14—C15—C16	−173.70 (14)
C31—Ru1—Ru3—C36	11.10 (9)	C19—C14—C15—C28	177.78 (17)
P1—Ru1—Ru3—C36	−70.18 (7)	P2—C14—C15—C28	5.6 (3)
Ru2—Ru1—Ru3—C36	−89.29 (7)	C14—C15—C16—C17	1.0 (3)
C30—Ru1—Ru3—P3	−172.38 (7)	C28—C15—C16—C17	−178.40 (19)
C32—Ru1—Ru3—P3	−80.69 (6)	C15—C16—C17—C18	0.0 (3)
C31—Ru1—Ru3—P3	100.73 (6)	C16—C17—C18—C19	−0.3 (3)
P1—Ru1—Ru3—P3	19.45 (4)	C17—C18—C19—C14	−0.3 (3)
Ru2—Ru1—Ru3—P3	0.34 (3)	C15—C14—C19—C18	1.2 (3)
C30—Ru1—Ru3—Ru2	−172.72 (6)	P2—C14—C19—C18	173.59 (15)
C32—Ru1—Ru3—Ru2	−81.03 (5)	C14—P2—C20—C25	131.38 (14)
C31—Ru1—Ru3—Ru2	100.39 (6)	C13—P2—C20—C25	−121.42 (15)
P1—Ru1—Ru3—Ru2	19.11 (3)	Ru2—P2—C20—C25	5.38 (17)
C33—Ru2—Ru3—C38	−177.9 (2)	C14—P2—C20—C21	−50.36 (18)
C34—Ru2—Ru3—C38	−86.1 (2)	C13—P2—C20—C21	56.84 (17)
C35—Ru2—Ru3—C38	94.9 (2)	Ru2—P2—C20—C21	−176.36 (13)
P2—Ru2—Ru3—C38	16.5 (2)	C25—C20—C21—C22	−0.6 (3)
Ru1—Ru2—Ru3—C38	−4.5 (2)	P2—C20—C21—C22	−178.81 (15)
C33—Ru2—Ru3—C37	98.57 (8)	C25—C20—C21—C29	178.70 (19)
C34—Ru2—Ru3—C37	−169.65 (8)	P2—C20—C21—C29	0.5 (3)
C35—Ru2—Ru3—C37	11.38 (8)	C20—C21—C22—C23	0.5 (3)
P2—Ru2—Ru3—C37	−67.04 (6)	C29—C21—C22—C23	−178.9 (2)
Ru1—Ru2—Ru3—C37	−88.00 (6)	C21—C22—C23—C24	0.0 (3)
C33—Ru2—Ru3—C36	−81.92 (8)	C22—C23—C24—C25	−0.4 (3)
C34—Ru2—Ru3—C36	9.86 (8)	C23—C24—C25—C20	0.2 (3)
C35—Ru2—Ru3—C36	−169.11 (8)	C21—C20—C25—C24	0.2 (3)
P2—Ru2—Ru3—C36	112.47 (6)	P2—C20—C25—C24	178.59 (15)
Ru1—Ru2—Ru3—C36	91.52 (6)	C32—Ru1—C30—O1	−103 (9)
C33—Ru2—Ru3—P3	6.71 (6)	C31—Ru1—C30—O1	72 (9)
C34—Ru2—Ru3—P3	98.49 (6)	P1—Ru1—C30—O1	162 (9)
C35—Ru2—Ru3—P3	−80.48 (6)	Ru3—Ru1—C30—O1	−12 (9)
P2—Ru2—Ru3—P3	−158.90 (3)	Ru2—Ru1—C30—O1	−32 (10)
Ru1—Ru2—Ru3—P3	−179.853 (13)	C30—Ru1—C31—O2	30.3 (13)
C33—Ru2—Ru3—Ru1	−173.44 (6)	C32—Ru1—C31—O2	116.5 (13)
C34—Ru2—Ru3—Ru1	−81.65 (6)	P1—Ru1—C31—O2	−75.9 (13)
C35—Ru2—Ru3—Ru1	99.37 (5)	Ru3—Ru1—C31—O2	132.7 (13)
P2—Ru2—Ru3—Ru1	20.96 (3)	Ru2—Ru1—C31—O2	−168.3 (13)
C30—Ru1—P1—C7	−88.09 (9)	C30—Ru1—C32—O3	−26.6 (14)
C32—Ru1—P1—C7	178.69 (8)	C31—Ru1—C32—O3	−112.6 (15)
C31—Ru1—P1—C7	−0.20 (8)	P1—Ru1—C32—O3	79.9 (14)
Ru3—Ru1—P1—C7	79.86 (7)	Ru3—Ru1—C32—O3	−128.7 (14)
Ru2—Ru1—P1—C7	96.35 (6)	Ru2—Ru1—C32—O3	171.6 (14)
C30—Ru1—P1—C1	36.35 (9)	C34—Ru2—C33—O4	79 (6)
C32—Ru1—P1—C1	−56.87 (8)	C35—Ru2—C33—O4	−108 (6)
C31—Ru1—P1—C1	124.24 (9)	P2—Ru2—C33—O4	−18 (6)
Ru3—Ru1—P1—C1	−155.70 (6)	Ru3—Ru2—C33—O4	169 (100)
Ru2—Ru1—P1—C1	−139.21 (7)	Ru1—Ru2—C33—O4	147 (6)
C30—Ru1—P1—C13	155.03 (9)	C33—Ru2—C34—O5	−20.8 (15)
C32—Ru1—P1—C13	61.81 (8)	C35—Ru2—C34—O5	−117.7 (15)
C31—Ru1—P1—C13	−117.08 (9)	P2—Ru2—C34—O5	81.2 (15)
Ru3—Ru1—P1—C13	−37.02 (7)	Ru3—Ru2—C34—O5	−126.9 (15)
Ru2—Ru1—P1—C13	−20.53 (7)	Ru1—Ru2—C34—O5	173.4 (15)
C33—Ru2—P2—C14	−84.73 (8)	C33—Ru2—C35—O6	28.4 (15)
C34—Ru2—P2—C14	−177.93 (8)	C34—Ru2—C35—O6	125.5 (15)
C35—Ru2—P2—C14	4.15 (8)	P2—Ru2—C35—O6	−73.3 (15)
Ru3—Ru2—P2—C14	81.15 (6)	Ru3—Ru2—C35—O6	134.7 (15)
Ru1—Ru2—P2—C14	99.20 (6)	Ru1—Ru2—C35—O6	−166.3 (15)
C33—Ru2—P2—C20	39.67 (9)	C38—Ru3—C36—O7	−51.3 (15)
C34—Ru2—P2—C20	−53.53 (9)	C37—Ru3—C36—O7	−48 (3)
C35—Ru2—P2—C20	128.55 (8)	P3—Ru3—C36—O7	53.0 (15)
Ru3—Ru2—P2—C20	−154.44 (6)	Ru1—Ru3—C36—O7	−152.4 (15)
Ru1—Ru2—P2—C20	−136.40 (7)	Ru2—Ru3—C36—O7	147.4 (15)
C33—Ru2—P2—C13	158.65 (9)	C38—Ru3—C37—O8	39.4 (12)
C34—Ru2—P2—C13	65.45 (9)	C36—Ru3—C37—O8	36 (3)
C35—Ru2—P2—C13	−112.47 (8)	P3—Ru3—C37—O8	−64.9 (12)
Ru3—Ru2—P2—C13	−35.46 (7)	Ru1—Ru3—C37—O8	140.5 (12)
Ru1—Ru2—P2—C13	−17.42 (7)	Ru2—Ru3—C37—O8	−159.4 (12)
C38—Ru3—P3—O11	−134.82 (9)	C37—Ru3—C38—O9	5 (3)
C37—Ru3—P3—O11	−48.23 (8)	C36—Ru3—C38—O9	−175 (3)
C36—Ru3—P3—O11	133.59 (9)	P3—Ru3—C38—O9	96 (3)
Ru1—Ru3—P3—O11	43.34 (8)	Ru1—Ru3—C38—O9	−83 (3)
Ru2—Ru3—P3—O11	43.64 (6)	Ru2—Ru3—C38—O9	−79 (3)
C38—Ru3—P3—O10	104.49 (9)	P3—O10—C39—C40	69.4 (2)
C37—Ru3—P3—O10	−168.93 (8)	P3—O10—C39—C44	−112.13 (19)
C36—Ru3—P3—O10	12.90 (9)	C44—C39—C40—C41	1.8 (3)
Ru1—Ru3—P3—O10	−77.35 (7)	O10—C39—C40—C41	−179.8 (2)
Ru2—Ru3—P3—O10	−77.05 (6)	C39—C40—C41—C42	−1.3 (4)
C38—Ru3—P3—O12	−16.47 (9)	C40—C41—C42—C43	−0.1 (4)
C37—Ru3—P3—O12	70.12 (8)	C41—C42—C43—C44	1.2 (4)
C36—Ru3—P3—O12	−108.06 (9)	C42—C43—C44—C39	−0.7 (4)
Ru1—Ru3—P3—O12	161.70 (6)	C40—C39—C44—C43	−0.7 (3)
Ru2—Ru3—P3—O12	161.99 (6)	O10—C39—C44—C43	−179.2 (2)
O11—P3—O10—C39	41.17 (17)	P3—O11—C45—C50	101.4 (2)
O12—P3—O10—C39	−60.61 (16)	P3—O11—C45—C46	−84.0 (2)
Ru3—P3—O10—C39	169.50 (13)	C50—C45—C46—C47	−1.1 (3)
O10—P3—O11—C45	−101.64 (17)	O11—C45—C46—C47	−175.6 (2)
O12—P3—O11—C45	4.36 (18)	C45—C46—C47—C48	0.2 (4)
Ru3—P3—O11—C45	133.72 (15)	C46—C47—C48—C49	0.7 (4)
O11—P3—O12—C51	171.31 (14)	C47—C48—C49—C50	−0.6 (4)
O10—P3—O12—C51	−82.04 (15)	C46—C45—C50—C49	1.1 (3)
Ru3—P3—O12—C51	43.10 (16)	O11—C45—C50—C49	175.58 (19)
C7—P1—C1—C6	134.78 (14)	C48—C49—C50—C45	−0.3 (3)
C13—P1—C1—C6	−119.06 (15)	P3—O12—C51—C52	44.4 (2)
Ru1—P1—C1—C6	7.96 (16)	P3—O12—C51—C56	−136.63 (14)
C7—P1—C1—C2	−48.53 (18)	C56—C51—C52—C53	2.5 (3)
C13—P1—C1—C2	57.63 (17)	O12—C51—C52—C53	−178.57 (17)
Ru1—P1—C1—C2	−175.35 (13)	C51—C52—C53—C54	−1.4 (3)
C6—C1—C2—C3	0.6 (3)	C52—C53—C54—C55	−0.8 (3)
P1—C1—C2—C3	−176.01 (15)	C53—C54—C55—C56	2.0 (3)
C6—C1—C2—C26	−179.09 (19)	C52—C51—C56—C55	−1.3 (3)
P1—C1—C2—C26	4.3 (3)	O12—C51—C56—C55	179.73 (16)
C1—C2—C3—C4	−0.9 (3)	C54—C55—C56—C51	−1.0 (3)
C26—C2—C3—C4	178.8 (2)		

### Hydrogen-bond geometry (Å, º)

**Table:**

*D*—H···*A*	*D*—H	H···*A*	*D*···*A*	*D*—H···*A*
C42—H42*A*···O1^i^	0.95	2.52	3.384 (3)	151
C56—H56*A*···O8^ii^	0.95	2.59	3.343 (3)	136

Symmetry codes: (i) *x*, *y*−1, *z*; (ii) −*x*+1, −*y*, −*z*+1.
